# Preschool Expulsion Risk Factors: Teachers’ Ratings of Preschoolers’ Challenging Behaviors Vary by the Cooperativeness of their Parents

**DOI:** 10.1007/s11121-025-01838-3

**Published:** 2025-09-15

**Authors:** Courtney A. Zulauf-McCurdy, Rechele Brooks, Andrew N. Meltzoff

**Affiliations:** 1 Pritzker Department of Psychiatry and Behavioral Health, Ann & Robert H., Lurie Childrens Hospital of Chicago, Chicago, IL USA; 2 Institute for Learning & Brain Sciences, University of Washington, Seattle, WA USA; 3 Department of Psychology, University of Washington, Seattle, WA USA; 4 Psychiatry & Behavioral Sciences, Northwestern Feinberg School of Medicine, Chicago, IL USA

**Keywords:** Preschool, Expulsion, School discipline, Parent-teacher relations, Prevention

## Abstract

Preschool children in the United States are expelled at a rate of about 250 per day with a disproportionate number being Black children. There is a need to better understand how to prevent preschool expulsion. Using a random-assignment experiment, preschool teachers (*N* = 95; 92% female; 61% White) were assigned to one of eight conditions: child race (Black vs. White) $\times$ child gender (boy vs. girl) $\times$ parental cooperativity (cooperative vs. uncooperative). Teachers read two controlled vignettes: one about a child and one about that child’s parents. The child vignette described the child’s challenging classroom behavior (identical behavior for all children); the parent vignette described a subsequent meeting with the child’s parents (half of the parents were described as uncooperative with the teacher and half as cooperative). After each vignette, teachers were asked to complete sections of the Preschool Expulsion Risk Measure (PERM) to assess two known risk factors for preschool expulsion: (a) teachers’ perception of the disruptiveness of the child’s classroom behavior and (b) teachers’ feelings of hopelessness about changing the child’s behavior. Even though the child’s challenging behavior was controlled (by experimental design), teachers’ construal of the child’s behavior and teachers’ feelings of hopelessness towards the child were significantly influenced by the descriptions of parental cooperation. Variations in results by child race and child gender are also reported. Findings indicate that teachers’ perceptions of parents may be a particularly important factor to prevent children from preschool expulsion.

**Supplementary Information:**

The online version contains supplementary material available at 10.1007/s11121-025-01838-3.

About 250 preschool children are expelled from their classroom each day in the United States (U.S. Department of Education, 2016; Zeng et al., 2019). Nationwide data from the 2015–2016 and 2017–2018 school years indicated that rates of preschool expulsion are even higher than K-12 expulsion rates (Fabes et al., 2020; Meek et al., 2020). Children who are expelled from preschool are subsequently more likely to experience academic failure and to be in contact with the juvenile justice system (Rosenbaum, 2020). It has been argued by scientists and early childhood advocates that preschool children who are expelled are often entering a “cradle-to-prison pipeline”—such that the arc of children’s developmental trajectory is bent away from the school system and into the criminal justice system (e.g., Equal Justice Society, 2018). To reduce these early childhood disparities, it is critical to identify factors that protect against expulsion from preschool. Such work has implications for child developmental theory, public policy, and how we think about society, education, and children.

The preschool period is a developmental inflection point in which children are learning social, behavioral, and emotional skills that influence future academic and social success. Research has revealed that attendance in a preschool program is correlated with better educational, occupational, and social outcomes (Melhuish et al., 2008; Sammons et al., 2008). For example, a 25-year-longitudinal study of 1400 students in a federally funded preschool program (largely serving Black and low-income households) reported enduring benefits with the students ultimately having higher educational levels, incomes, socioeconomic status, and lower rates of substance abuse and legal problems compared to comparison peers who did not attend the program (Reynolds et al., 2011). When a preschooler is expelled, he or she is denied access to early educational environments that have been shown to promote positive long-term outcomes—with expulsions often befalling the very children who are in the most need of preschool experience before they enter the formal schooling pipeline.

## Preschool Expulsion Risk Factors

Following Gilliam’s (2005) landmark analysis of patterns of preschool expulsion, researchers have deepened efforts to understand what factors influence a preschool teacher’s decision to remove a child from their classroom (Sabol et al., 2021; Zinsser et al., 2022). Most research to date on preschool children has explored teachers’ implicit biases in decision-making about expulsions (Meltzoff & Gilliam, 2024; for older children see Goff et al., 2014; Okonofua & Eberhardt, 2015; Todd et al., 2016). For example, in a study focusing on preschool teachers, Gilliam, Maupin and Reyes et al., (2016a; Study 1) conducted a study in which teachers watched short video clips of children in a classroom and were told that the videos “may or may not contain challenging behavior.” The teachers were asked to press a button when they first saw the challenging behavior. Despite the absence of challenging behavior in the videos (by experimental design), the results showed that preschool teachers looked longer at the Black children compared to the White children, suggesting that teachers presumed that the Black children were more likely to display challenging behavior.

### Teachers’ Perceptions of Parents

Less effort has been focused on understanding whether preschool teachers’ attitudes and potential bias towards *parents* may influence a child’s risk for expulsion. Prior work suggests that preschool teachers’ decision making about how to respond to child behavior may be influenced by their perception of the cooperativity of the children’s parents. Martin et al. (2018) used a qualitative approach—in-depth interviews with preschool teachers—to explore teachers’ recollections about their decision-making leading up to the expulsion of a preschooler. According to teachers’ recollections, when a teacher was faced with challenging behavior they first searched for contextual causes in the classroom (e.g., classroom transitions cause tantrums). If the teacher was unable to find such a contextual cause, they tended to turn their focus to the child (e.g., blaming the child) and, further along in the process, to the parents (e.g., the attribution that they are being uncooperative). When and if teachers shifted the cause of the behavior to the parents, expulsion was more likely to occur.

Here, we focus on this understudied but potentially important linkage between a teacher’s perception of a child’s parents and the teacher’s construal of that child’s behavior in the classroom. Moving beyond retrospective and correlational approaches, we designed a random-assignment experimental protocol to measure the direct impact of parental cooperation on teachers’ perceptions of child behavior. The key element was to control for the child’s behavior and manipulate the child’s race, child’s gender, and level of parental cooperativity through two controlled vignettes that varied in a systematic fashion.

A few studies have explored the relation between teachers’ perceptions of parents and a child’s risk for expulsion. In a retrospective study of preschool teachers’ recollections, it was found that teachers who had requested an expulsion in the past year endorsed more negative perceptions of parents compared to teachers who had not requested an expulsion (Zulauf & Zinsser, 2019). Similarly, teachers’ ratings of a child’s risk for future expulsion were influenced by whether teachers perceived themselves as having a high-quality relationship with the parents (Zulauf-McCurdy & Zinsser, 2021). Gilliam, Maupin and Reyes et al., (2016a, b; Study 2) presented preschool teachers with a written vignette describing a child displaying challenging behavior in the classroom and randomly assigned half the teachers to receive information describing the child as living in a single-parent (mother) household. Teachers who were provided the family background information perceived the child’s challenging behavior as more severe and felt more hopeless about changing the child behavior than teachers who did not receive this background information.

## Protective Factors for Preschool Expulsion

A better understanding of protective factors is needed to guide interventions to help reduce the number of preschool expulsions. Many efforts to decrease expulsion have focused on system-wide changes such as providing early childhood mental health consultations (Gilliam, Maupin, & Reyes, 2016a; Reyes & Gilliam, 2021) or increasing equity-focused Positive Behavioral Interventions and Supports (PBIS; Shepley & Grisham-Brown, 2019). However, to date, most of these interventions face implementation challenges across diverse preschool settings and require significant funding to consistently implement (Meek et al., 2020). Therefore, there is a need to identify potential strategies to protect children from expulsion that are possible in a greater number of preschool settings at lower costs.

Parent-teacher relationships can be a powerful factor in supporting children with challenging behavior and therefore are a potential protective factor for expulsion. The potential value of connecting the experiences between home and preschool was outlined by Bronfenbrenner (1986) and emphasized by Epstein (1995) referring to it as “spheres of influence” in a child’s life. Specifically, through fostering strong connections between homes and schools, teachers and parents at all grade levels can work together to promote better social, behavioral, and academic outcomes for children (Serpell & Mashburn, 2012; Sheridan et al., 2017; Smith et al., 2019). These connections may be especially important in preschool, because it has been reported that when there is a weak or poor parent-teacher relationship, this can be detrimental to student success (Martin et al., 2018; Zulauf & Zinsser, 2019). The present study advances our understanding about whether *teachers’ perceptions of parents as cooperative* agents may be used to protect children from preschool expulsion.

### Present Study

Our principal goal was to address the following research question: Are teachers’ ratings of child behavior and their attitudes towards the child’s behavior influenced by *parental* cooperation and does this influence hold across child race and gender? It was hypothesized that providing preschool teachers with (controlled) information about the cooperativity of parents would systematically influence their beliefs and attitudes about the child. However, given the paucity of research about the risk for preschool expulsion, we did not hold specific hypotheses about potential interactions by child race and gender. Therefore, this study can best be classified as exploratory rather than confirmatory experimental work.

## Method

### Participants

Our prespecified inclusion criteria were that participants had to be a lead teacher serving children between the ages of 3 to 5 years old in a group-based school or educational setting that was credentialed or licensed. Teachers were not eligible if employed by a home-based early childhood provider. Only lead teachers were recruited because they are typically instrumental in the decision-making for preschool expulsions. Potential participants were obtained through professional network listservs and school partnerships in Washington. All participants gave informed consent online in accordance with the Institutional Review Board at the authors’ institution (IRB # STUDY00011241). We preregistered the design plan, sampling plan, and variables at the Open Science Framework.

Out of 109 people who accessed the online screener, 96 teachers met the preestablished inclusion criteria, and 95 of them completed the study. Teachers had an average of 12.04 years of experience working in preschool settings. At the preschool settings in which teachers taught, the proportion of parents who paid tuition varied as follows: all parents paid tuition (24%), some parents paid tuition (22%), and no parents paid tuition (54%). See Table 1 for additional teacher demographics in the analytic sample of 95. Each participant, upon completion of their online responses, received a $15 gift card. Responses were collected over an approximately 6-month period (July 1, 2021 to December 31, 2021) to ensure that all teachers completed the study during the same part of the school year (first half of the school year). Because of slower than expected recruitment (during the COVID-19 pandemic), we were not able to reach our preregistration goal of 240 participants (see “Limitations and Future Directions”).

**Table 1:** Teachers’ descriptive statistics for randomly assigned groups of parental cooperativity and for the full analytic sample

	Group^a^		Total	
	Cooperative	Uncooperative		
Teacher demographics	*n* = 49	*n* = 46	*N* = 95	Percent
Gender of teacher				
Female	45	43	88	92.63
Male	2	1	3	3.16
Non-binary or self-described	2	2	4	4.21
Race of teacher				
White	29	29	58	61.05
Black/African American	7	7	14	14.74
Asian	4	0	4	4.21
More than one race	3	2	5	5.26
Other racial identities^b^	5	6	11	11.58
Did not report	1	2	3	3.16
Ethnicity of teacher				
Hispanic or Latinx	8	12	20	21.05
Education level of teacher				
High school graduate	12	9	21	22.11
Associate degree	12	16	28	29.47
Bachelor degree	16	13	29	30.53
Master’s degree	9	8	17	17.89

^a^We tested whether any of the teacher demographics (column 1) varied as a function of the two randomly assigned groups: cooperative (column 2) vs. uncooperative (column 3). None of the chi-square tests was significant

^b^Of the 11 teachers who selected “other” racial identities, ten described themselves as Hispanic, Latinx, or Mexican ethnicity (thus they are also in the row showing “20” in the table) and one identified as Slavic

### Materials and Procedures

**Fig. 1:**
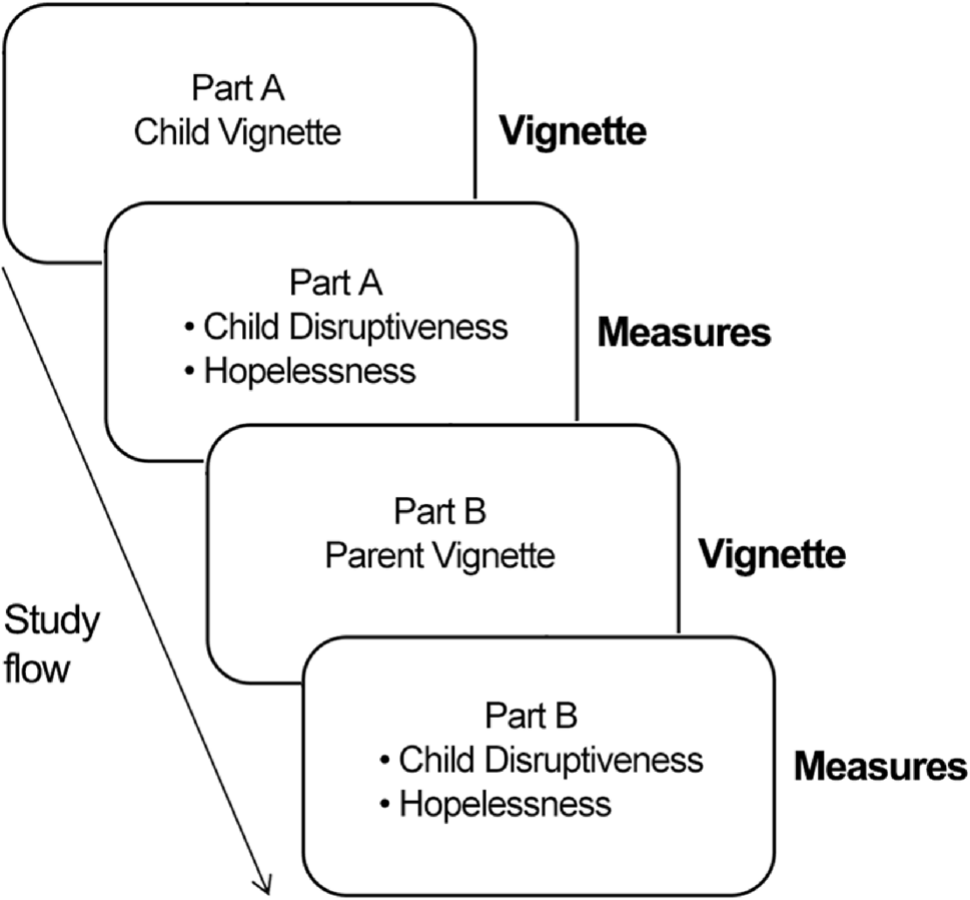
Flow of the study. The measures in Part A and Part B were teacher ratings of the child. These measures were gathered both before and after teachers read the parent vignette about parental cooperativity (repeated measures)

All study materials were constructed and posted online in Qualtrics. Figure 1 outlines the study flow. The entire study was done in one sitting, with teachers first completing Part A (child vignette and measures) and then immediately completing Part B (parent vignette and measures). It typically took teachers 12–13 min (median = 12.35 min) to complete the online study.

Each teacher was randomly assigned to the first experimental condition (Part A) as follows (described in more detail below): (a) a stereotypical Black boy name (DeShawn), (b) a stereotypical White boy name (Jake), (c) a stereotypical Black girl name (Latoya), or (d) a stereotypical White girl name (Emily). These names were selected from well-accepted prior research (e.g., Bertrand & Mullainathan, 2004; Okonofua & Eberhardt, 2015) and were identical to those used in the Gilliam, Maupin and Reyes et al., (2016a) study. After completion of Part A, the teachers were randomly assigned to the second experimental condition to systematically vary whether or not the parents were cooperative. Therefore, there were a total of eight conditions (Black boy with cooperative parents, Black boy with uncooperative parents, etc.; for more details about all eight experimental conditions, see Supplementary Information, Section 1).

After ensuring eligibility and consent, the procedure unfolded as follows. Teachers were instructed that they were going to read short stories about a preschool child and that they would be asked to answer questions about how they would interact with the child and support the child’s behavior.

During Part A, all teachers read a standardized vignette describing a preschool child who exhibited challenging classroom behavior (drawn from Gilliam, Maupin, Reyes et al., 2016a Study 2; see Supplementary Information, Section 2, for verbatim child vignette). Teachers were asked to imagine that the child was in their classroom. Critically, the exact description of the child’s behavior was controlled and remained identical for all teachers; in the child vignette, the only word changed per condition was the name of the child. Immediately after reading the child vignette, teachers completed Part A measures assessing teachers’ ratings of (a) the disruptiveness of the child’s behavior and (b) hopelessness about changing the child’s behavior. We purposely assessed each teacher’s ratings immediately after reading the child vignette to examine ratings when teachers knew about the child’s assumed race and gender (based on the stereotypical name) but before they knew about parental cooperativity (see Fig. 1).

Next in Part B, teachers were asked to imagine that they brought the child’s parents into the classroom so they could speak to them about the child’s behavior (see Supplementary Information, Section 3, for verbatim parent vignette). Prior to this vignette, teachers were not aware that they would be receiving a story about the parents. In the parent vignette, the child’s name remained the same as Part A. This parent vignette was created specifically for this study and was based on previous qualitative work with teachers and parents about expulsion. In Part B, teachers were randomly assigned to receive a controlled vignette either describing: (a) *cooperative* parents (parents acknowledged the teacher’s concern and asked how to support their child) or (b) *uncooperative* parents (parents denied the teacher’s concern and did not listen to any recommendations). After reading the parent vignette, teachers completed Part B measures about their ratings of the child (identical to Part A measures). This assessment, after reading the parent vignette, allowed us to examine the effect of parental cooperativity on teacher ratings of the child.

### Measures and Analysis Plan

#### Teachers’ Ratings of Classroom Disruption and Hopelessness

In both Part A and Part B, teachers rated 6 items from the Preschool Expulsion Risk Measure (PERM; Gilliam & Reyes, 2018) about their views of the child’s behavior. Each item had a five-point Likert scale from 1 (*strongly disagree*) to 5 (*strongly agree*). The 6 items capture two factors of the PERM, both directly related to a child’s expulsion risk: (a) the degree to which the teacher perceives that the child’s behavior causes *classroom disruption* (three items, e.g., “This child’s classroom behaviors interfere with my ability to teach effectively”) and (b) the degree to which the teacher feels *hopelessness* that anything can be done to improve the child’s behavior (three items, e.g., “This child’s classroom behaviors are not likely to improve significantly”). To respect teachers’ busy schedules, the online questionnaire was kept short by omitting two additional factors of the PERM (these items were also not used by Gilliam, Maupin, Reyes et al., 2016a, see Supplementary Information, Section 4, for further discussion).

The PERM hopelessness factor includes an item asking about whether the parents will “help” with the child, and our experimental manipulation also included a description of the parents offering to “help” with their child. To address potential concerns about using the same word in the manipulation as in the outcome measure, we created two versions of the hopelessness measure: the standard version and a revised version that omitted the single item mentioning parent helpfulness. Analyses revealed that the results with the standard and revised versions were substantively the same in all important respects (see Supplementary Information, Section 5). Given this, we therefore present the results with the revised version to conservatively test the ratings without the use of duplicate wording.

In the present study, both the classroom disruption and hopelessness measures showed good internal consistency in Part A (*ɑ* = .82 and *ɑ* = .78, respectively) and Part B (*ɑ* = .84 and *ɑ* = .77, respectively).

#### Analysis Plan

First, the data were examined, and there was no evidence for outliers or statistical assumption violations. Second, there were no missing data, because all 95 teachers completed all Part A and Part B measures. Third, we conducted preliminary analyses to explore whether there were effects of teacher demographics (race, ethnicity, education, years of teaching experience) or preschool tuition (tuition charged to none, some, or all parents) on the outcome scores (teacher ratings of classroom disruption and hopelessness). To accomplish this, we used analyses of variance (ANOVAs) for categorical variables and Pearson correlation for the continuous variable. We found no significant effects (all *p*-values > 0.05), and therefore, for parsimony and conservation of degrees of freedom, we collapsed across these factors in the main analyses. Additional ANCOVA analyses were conducted with the teacher demographic information and preschool tuition as covariates; with effects of child’s race, child’s gender, and parental cooperativity as between-subject factors; and with part of test (A vs. B) as a within-subject factor, which yielded substantively similar findings as the main results (see Supplementary Information, Section 7).

In the final models, teacher ratings of classroom disruption and hopelessness were separately analyzed with a mixed ANOVA with child’s race, child’s gender, and parental cooperativity as the between-subjects factors, and part (Part A vs. Part B) as the within-subject factor. Based on the literature with controlled vignettes of this type (e.g., Gilliam, Maupin, Reyes et al., 2016a Okonofua & Eberhart, 2015), a priori power analysis showed that a sample size of *N* = 80 would afford 80% power to detect the predicted effect of parental cooperativity (expected medium effect size) and its potential two-way and three-way interactions in a mixed design (using both between- and within-subjects factors, e.g., parental cooperativity and part, parental cooperativity by race and part) as determined with G*Power (Faul et al., 2007). In our present study, the analytic sample consisted of *N* = 95 participants. The power analysis also indicated that a *N* = 128 would be needed for exploratory analyses of four-way interactions (e.g., the interaction of child race, child gender, parental cooperativity, and part). Given that the present study was somewhat underpowered with respect to higher-order interactions, we also ran analyses with a simpler factorial model as a check of sensitivity and robustness (Supplementary Information, Section 8). These supplemental analyses yielded significant results highly similar to the full factorial model, so we present the full factorial model for completeness. All analyses were conducted with IBM SPSS Statistics (Version 28).

## Results

Table 2 presents the descriptive statistics for each measure. The correlations among the measures are presented in Table S1 (Supplementary Information, Section 4). For example, classroom disruption and hopelessness were significantly correlated in Part A, *r* = .35, *p* < 0.001.

**Table 2:** Teacher ratings of classroom disruption and hopelessness as function of Part A versus B

	Part A		Part B	
Parental cooperativity	Mean	*(SD*)	Mean	*(SD*)
	Classroom disruptiveness			
Cooperative	3.73*	(0.95)	3.50*	(1.07)
Uncooperative	3.67	(0.84)	3.73	(0.72)
	Hopelessness			
Cooperative	1.78	(0.95)	1.67	(0.88)
Uncooperative	1.58*	(0.74)	2.08*	(1.00)

Part A measures were taken before teachers read the vignette about parental cooperativeness; Part B measures were taken after the teachers read the vignette about parental cooperativeness

*p < 0.01 difference between Part A and Part B for effects that share the same line

### Classroom Disruption Measure

We conducted a 2 (child’s race: Black vs. White) $\times$ 2 (child’s gender: boy vs. girl) $\times$ 2 (parental cooperativity: cooperative vs. uncooperative) $\times$ 2 (part: A vs. B) ANOVA on teachers’ ratings of *classroom disruption*. There were no significant main effects or three or four-way interactions, but several two-way interactions were significant (Table 3).

**Table 3:** Repeated-measures analysis (ANOVA) with criterion of classroom disruptiveness

Measure	*F*(1, 87)	Partial ${\eta }^{2}$
Within subjects		
Part (P)	2.55	.03
Child gender (G) $\times$ P	0.02	.00
Child race (R) $\times$ P	5.01*	.05
Parental cooperativity (C) $\times$ P	8.58**	.09
G $\times$ R $\times$ P	0.16	.00
G $\times$ C $\times$ P	1.19	.01
R $\times$ C $\times$ P	1.70	.02
G $\times$ R $\times$ C $\times$ P	1.86	.02
Between subjects		
Gender	0.34	.00
Race	0.50	.01
Parental cooperativity	0.30	.00
G $\times$ R	0.92	.01
G $\times$ C	1.70	.02
R $\times$ C	0.67	.01
G $\times$ R $\times$ C	0.10	.00

*p < 0.05. **p < 0.01

The two-way interaction of Parental Cooperativity $\times$ Part was significant, *F*(1, 87) = 8.58, *p* = 0.004, partial ${\eta }^{2}$  = .09. Breaking down this interaction (Table 2), we found that teachers’ ratings of children’s *classroom disruption* significantly decreased from Part A (child vignette) to Part B (parent vignette) for the teachers who read about the *cooperative* parents (Part A: *M* = 3.73 vs. Part B: *M* = 3.50), *t*(48) = 3.26, *p* = 0.002, Cohen’s *d* = 0.47. For teachers who read about the uncooperative parents, ratings of classroom disruption did not significantly change from Part A to Part B (Part A: *M* = 3.67 vs. Part B: *M* = 3.73), *t*(45) = .79, *p* = 0.43, Cohen’s *d* = 0.12).

The two-way interaction of Child Race $\times$ Part was also significant, *F*(1, 87) = 5.01, *p* = 0.028, partial ${\eta }^{2}$  = .05. Breaking down this interaction, we found that for the White children, teachers’ ratings of classroom disruption significantly *decreased* from Part A (*M* = 3.83, *SD* = 0.89) to Part B (*M* = 3.63, *SD* = 0.96), *t*(48) = 2.79, *p* = 0.008, Cohen’s *d* = 0.40. The mean ratings for the Black children did not significantly change from Part A (*M* = 3.57) to Part B (*M* = 3.59), *t*(45) = 0.28, *p* = 0.77, Cohen’s *d* = 0.04.

### Hopelessness Measure

For the hopelessness measure, we conducted the same ANOVAs described above. There were no significant three- or four-way interactions. There were significant main effects and a significant two-way interaction (Table 4).

**Table 4:** Repeated-measures analysis (ANOVA) using criterion of hopelessness

Measure	*F*(1, 87)	Partial ${\eta }^{2}$
Within subjects		
Part (P)	8.34**	.09
Child gender (G) $\times$ P	0.04	.00
Child race (R) $\times$ P	0.39	.00
Parental cooperativity (C) $\times$ P	18.22***	.17
G $\times$ R $\times$ P	2.07	.02
G $\times$ C $\times$ P	0.03	.00
R $\times$ C $\times$ P	1.80	.02
G $\times$ R $\times$ C $\times$ P	0.41	.00
Between subjects		
Child gender	6.65*	.07
Child race	0.44	.01
Parental cooperativity	0.26	.00
G $\times$ R	0.53	.01
G $\times$ C	0.20	.00
R $\times$ C	0.59	.01
G $\times$ R $\times$ C	0.50	.01

*p < 0.05. **p < 0.01. ***p < 0.001

The main effect for part (A vs. B) was significant, *F*(1, 87) = 8.34, *p* = .005, partial ${\eta }^{2}$ = .09, with ratings of hopelessness increasing from Part A (*M* = 1.68, *SD* = 0.85) to Part B (*M* = 1.87, *SD* = 0.95). Importantly, this effect was moderated by parental cooperativity, as shown by the significant interaction between part and parental cooperativity, *F*(1, 87) = 18.22, *p* = 0.00005, partial ${\eta }^{2}$^2^ = .17. Breaking down this interaction (Table 2), we found that ratings of hopelessness about changing the behavior of the child significantly *increased* for teachers who read about the *un*cooperative parents (Part A: *M* = 1.58 vs. Part B: *M* = 2.08), *t*(45) = 4.74, *p* = 0.00002, Cohen’s *d* = 0.70. Ratings of hopelessness did not significantly change for teachers who read about the cooperative parents (Part A: *M* = 1.78 vs. Part B: *M* = 1.67), *t*(48) = −1.08, *p* = 0.285, Cohen’s *d* =  − 0.15.

The main effect of child gender was significant, *F*(1, 87) = 6.65, *p* = 0.012, partial ${\eta }^{2}$ = .07. Preschool teachers endorsed stronger feelings of hopelessness after reading about the challenging behavior of girls (*M* = 1.98, *SD* = 0.97) compared to the identical description of challenging behavior of boys (*M* = 1.55, *SD* = 0.56).

## Discussion

Although the described behavior of the child was controlled and remained constant, we found that the experimentally manipulated description of parental cooperation influenced preschool teachers’ perceptions and attitudes about a child. When teachers were presented with a description of the child’s parents being cooperative, their ratings of classroom disruption decreased. When teachers read a description of a child’s parents being uncooperative, their feelings of hopelessness about the child’s capacity to change increased. The findings indicate with moderate effect sizes that providing preschool teachers with information that a child’s *parents* may cooperate (in supporting the child’s behavior) positively influences known outcomes in teachers that are related to preschool expulsion risk (i.e., teachers’ perception of classroom disruptiveness). There were also weak results for child race and gender (i.e., small effect sizes), see the discussion below for more details.

### Parental Cooperation Influences Teachers’ Perceptions of Children’s Disruption in the Classroom

The present study built upon Gilliam, Maupin, Reyes et al. and’s (2016a) work of presenting preschool teachers with a written vignette about a child’s behavior (Part A of this study). We went beyond this work by adding a vignette about parental cooperativeness (Part B of this study). Teachers were randomly assigned to receive a vignette that either described the parents as cooperative or uncooperative. The first novel finding in this study was that teachers who read a vignette describing the parents as cooperative significantly decreased their ratings of the child’s disruptiveness (from Part A to Part B), whereas teachers who read a vignette about the parents being uncooperative did not. These findings are notable because all teachers received identical information about the child’s *actual* behavior (by experimental design), suggesting that teachers’ construal of preschoolers’ behavior are influenced by descriptions of parents as cooperative.

This finding is consistent with prior research indicating that preschool teachers’ ratings of child behavior are subjective, malleable, and based on a wider context than the child’s actual behavior alone. For example, a correlational study of Head Start teachers found that teachers who reported positive relationships with a child’s parents viewed that child as having fewer behavioral concerns than children of parents with whom the teacher had a more negative relationship (Serpell & Mashburn, 2012). Research, policy, and practice have emphasized strong parent-teacher relationships, built by communication and cooperation, as a key factor in promoting positive child outcomes (e.g., Sheridan et al., 2019; Smith et al., 2020). Despite this literature, few studies have measured the impact of experimentally varying parental cooperation on teachers’ perceptions of the child, and, most importantly, no previous studies have done this in preschool. The present findings suggest that supporting preschool teachers in seeing parents as cooperative and willing to help the teacher address a child’s potentially disruptive classroom behavior may be a protective factor against preschool expulsion. Specifically, our findings suggest that if preschool teachers talk to parents about a child’s classroom behavior, and the parents are perceived as cooperative, the teachers view the child’s behavior as less disruptive, potentially lowering the child’s risk for expulsion.

### Parental Lack of Cooperation Influences Teachers’ Hopelessness Towards Children

Another novel result concerns the significant effect of parental cooperation on teachers’ feelings of hopelessness about the child’s potential to change their behavior. Teachers who read a vignette describing the parents as *un*cooperative significantly increased their ratings of hopelessness (from Part A to Part B), whereas teachers who read a vignette about the parents being cooperative did not. These findings fit with previous qualitative work by Martin et al. (2018) in which they outlined a potential pathway to preschool expulsion. Within this pathway, when preschool teachers are faced with challenging behavior that they cannot modify, teachers shift their focus from the preschooler to the parent—and at this point, expulsion becomes more likely (Martin et al., 2018).

We thus see that preschool expulsion is not solely a child problem, but a result of a series of adult evaluations, decisions, and actions that are influenced by a wider context. Because most preschoolers are not capable of deeply explaining their thoughts, emotions, and behavioral motivations (e.g., Moll & Meltzoff, 2011), it is particularly useful for parents and teachers to work together. Parents often have insights into their children’s current stressors and behaviors and therefore can be an essential source for teachers to better understand the child’s behavior in school. Prior research suggests that if teachers hold a negative perception towards the parents, this may hinder how parents and teachers work together to support the child’s needs (Zulauf & Zinsser, 2019; Zulauf-McCurdy & Zinsser, 2022). Thus, finding ways to support preschool teachers’ positive perceptions of parents, and especially whether teachers believe they will be able to work with the parents to address the child’s challenging behaviors, may be a lever for interrupting the pathway to preschool expulsion (see “Practical Implications” below).

Because feelings of hopelessness about one’s capacity to improve a child’s behavior is itself malleable, our findings suggest a potential point of intervention. Based on the current findings, this will be especially important when teachers perceive parents as uncooperative. Teachers and parents may change their attitudes toward each other if they are able to increase opportunities for positive interactions to align their goals (Dasgupta, 2013) and to see each other as “like me” (Meltzoff, 2013), pulling in the same direction.

### Preschool Teachers’ Perceptions as a Function of Children’s Race and Gender

The effects of children’s race and gender yielded small effect sizes, unlike the main experimental manipulation of parental cooperativity, which had moderate effect sizes. Future work with larger samples of preschool teachers is needed to examine the possible effects of child race and gender on teachers’ perceptions. Topics related to the finding with race and classroom disruptiveness could include exploration of teachers’ racial bias and beliefs about behavioral change in Black and White children (Gilliam, Maupin, Reyes et al., 2016b; Starck et al., 2020). In addition, the finding about child gender points to exploring teachers’ feelings of hopelessness about the challenging behavior of girls in the preschool classroom, an understudied phenomenon.

### Practical Implications

The present study indicates that positive information about parents can influence preschool teachers’ interpretation of children’s challenging behaviors. Given the need to identify feasible interventions to protect children from preschool expulsion, our results suggest a potential intervention point regarding teachers’ perceptions of parental cooperativeness.

Scientists and preschool teachers alike realize that coordination and alignment between home and school can be especially important in promoting positive outcomes for young children (Lee et al., 2020). However, to date, little work has been done to target teachers’ perceptions of parents in situations where they feel that parents are being uncooperative (Sheridan et al., 2019). These situations may occur at various points throughout the school year, suggesting multiple windows for intervention. For example, teachers’ initial opportunity to get a sense of parental cooperativity may occur at the beginning of the year during the first parent-teacher conference, or during the first few months of the year when teachers are hoping to see parental engagement (i.e., parents volunteering in class, parents responding to teacher questions). It may also occur once teachers face challenging behavior and start to search for sources to “explain” such behaviors (Martin et al., 2018) or may reoccur when the teachers reach out to parents to discuss their concerns. Research indicates that by the time teachers reach out to parents about their concerns, it is often too late (Zulauf-McCurdy & Zinsser, 2022), indicating a need to intervene earlier in the process. Therefore, it may be best to find ways to improve teachers’ perceptions of parental cooperativity prior to the teachers experiencing the challenging behavior in the preschool classroom. Suggestions for supporting teachers to reach this goal include a combination of helping teachers take the perspectives of parents, creating more face-to-face contact with parents, and/or preparing teachers at the start of the school year to speak to parents in ways that promote collaboration (Kalla & Broockman, 2020).

Importantly, our findings suggest that conveying parents’ willingness to be cooperative with the preschool teacher reduces teachers’ concerns about the child’s disruptive behavior (a known risk factor for preschool expulsions), and crucially, it does so for both Black and White children. Although teachers’ perceptions of parents could be influenced by implicit racial bias towards Black families, the goal of targeting teachers’ perceptions of parental cooperation, rather than of reducing racial bias more generally, may be a more tractable point of intervention. The literature on early childhood mental health consultation indicates that providing direct support to teachers who are faced with handling a child’s challenging behavior serves to decrease suspension and expulsion (Gilliam, Maupin, & Reyes, 2016a, ; Reyes & Gilliam, 2021). This model may offer a starting point for developing interventions to support teachers working with parents whom they view as uncooperative or challenging. Enhancing preschool teachers’ ability to work with parents promises to be a useful building block for interventions designed to protect children from preschool expulsion. Moreover, research by Schock and Jeon (2021) suggests that providing well-supported working environments for preschool teachers can have a positive effect on teacher attitudes and work quality, which may facilitate greater actual or perceived support from families.

### Limitations and Future Directions

This experiment has several strengths (including random assignment of preschool teachers to conditions and controlled vignettes), but it is not without limitations. First, given that the preschool teachers in our experiment were presented with vignettes on a computer, it is unclear whether this reflected how teachers would feel and behave in real life. In the real-world, preschool classrooms are chaotic, busy, and stressful (Schonert-Reichl et al., 2015) potentially causing teachers to fall back on more deeply ingrained, implicit beliefs and attitudes (e.g., stereotypes and biases) that may not be under conscious control (Meltzoff & Gilliam, 2024).

Second, given an increase in societal sensitivities about preschool expulsion patterns, teachers may have become aware of the study aims while completing the study. Third, the teachers were instructed to imagine that they were meeting with the parents and that the parents showed up. In everyday practice, teachers interact with parents in several ways, and it does not always result in an in-person meeting. Fourth, we did not evaluate the durability of the parental cooperation effect. Many interventions, especially those designed to ameliorate the negative effects of bias, have the limitation of being short-lived at best (e.g., Paluck et al., 2021), and the observed effects after exposing preschool teachers to a vignette about parental cooperation may also be short lived (although the real-life possibility of *distributed* positive encounters between a preschool teacher and cooperative parents may increase durability). Fifth, we acknowledge that our sample size was smaller than anticipated (the study took place during the COVID-19 pandemic). Although we found some effects for gender and race, the effect sizes were small and replication attempts in additional samples is warranted. Further, a larger *N* would be required to have adequate power for comprehensive tests of intersectionality—that is, how the race and gender of children, parents, and teachers, as well as levels of parental cooperation, may all interact to predict a child’s risk for expulsion. Given that previous work has found that teacher and child race, and the match/mismatch between them, may predict preschool expulsion (Gilliam, Maupin, Reyes et al., 2016b; Wymer et al., 2022), future work with larger *N*s are desirable to further examine interactions with parental cooperativity.

Finally, more experimental work is needed to investigate the specific mechanisms that connect preschool teachers’ *perceptions* of parental cooperation with teachers’ *reactions* to children’s behavior in the real world. For example, the current study did not assess either implicit or explicit racial bias, but in the real world, teachers’ stress level and/or fear of accountability (two other factors of the PERM) may interact with racial biases in stressful moments when children are displaying challenging behavior in the classroom. Thus, we acknowledge that there may be questions about the clinical meaningfulness of the results reported here and endorse the idea that large *N* studies with randomly assigned experimental interventions with preschool teachers in real classrooms are needed to equip schools and policymakers with more comprehensive information to address the issue of preschool expulsion.

## Conclusion

Young children are being expelled from their preschool classrooms at undesirable rates. Preschool classrooms are the settings that are supposed to inspire youngsters’ curiosity for learning and prepare children for formal education. Preschool expulsions can set off a cascade of negative events for children and families. The present study suggests that preschool teachers’ judgments about *parental* cooperation may be a relevant part of the preschool expulsion puzzle. This paper aims to be a catalyst for further research and practice aimed at identifying ways to support preschool teachers in their work with parents, and preparing both teachers and parents to cooperate on the common goal of maximizing the child’s well-being and future success. This is important for supporting children during their initial encounters with their first societal institution, the educational system. School is an institution in which children spend a good deal of time before leaving home to independently function in society. For reasons of educational opportunity, valuing our children, and societal fairness, it is important to find ways to substantially reduce the risk of preschool expulsions.

## Supplementary Information

Below is the link to the electronic supplementary material.

Supplementary Material 1 (DOCX 242 KB)

## Data Availability

Data are available from the first author upon reasonable request. The analytic code necessary to reproduce the analyses presented in this paper is available from the first author. The materials necessary (vignettes) to attempt to replicate the findings presented here are publicly accessible (Supplemental Materials).
