# Supplementary material for: Preschool Expulsion Risk Factors: Teachers’ Ratings of Preschoolers’ Challenging Behaviors Vary by the Cooperativeness of their Parents
Source: Prev Sci. 2025 Sep 15;26(7):1010–20. doi: 10.1007/s11121-025-01838-3 (PMC12465125; doi:10.1007/s11121-025-01838-3)
Supplement: Supplementary file 1 — Supplementary Material 1 (DOCX 242 KB) [file 11121_2025_1838_MOESM1_ESM.docx]

**Supplementary Information for**

“Preschool Expulsion Risk Factors: Teachers’ Ratings of Preschoolers’ Challenging Behaviors Vary by the Cooperativeness of their Parents”

*Prevention Science*

**1. Conditions**

Prior to starting the study, preschool teachers were randomly assigned to one of eight experimental conditions: (1) Black boy with uncooperative parents; (2) Black girl with uncooperative parents; (3) White girl with uncooperative parents; (4) White boy with uncooperative parents; (5) Black boy with cooperative parents; (6) Black girl with cooperative parents; (7) White girl with cooperative parents; (8) White boy with cooperative parents. The assigned child’s race and gender were depicted throughout the study by providing the child’s name in both the child (Part A) and parent (Part B) vignettes [Jake /DeShawn /Emily /Latoya]. In Part A, teachers read a short vignette about the child’s challenging classroom behaviors. In Part B, teachers read a parent vignette describing the same child’s *parents* as being cooperative or uncooperative. See main text for temporal flow of study, Figure 1.

**2. Part A: Child Vignette**

The child vignette was drawn from Gilliam et al. (2016), with less than 25 words removed for use in the current study. One child’s name, from the four names shown within [square brackets] below, was used for any one teacher. Single words and phrases omitted from the Gilliam et al. (2016) vignette are shown in *{italics}* below.

***2.1. Verbatim Content of the Child Vignette***

I would like you to imagine that the following child is in your classroom.

[Jake /DeShawn /Emily /Latoya] *{is a four-year-old in your classroom with}* has unpredictable and challenging behaviors. [Jake /DeShawn /Emily /Latoya] has daily difficulties following instructions and waiting their turn, and their challenging behaviors escalate quickly. When other children are playing with toys they are interested in, [Jake /DeShawn /Emily /Latoya] yanks the toys away from them. When asked to return the toy and wait their turn, [Jake /DeShawn /Emily /Latoya] often pushes and hits either you or the other child. During circle time activities, [Jake /DeShawn /Emily /Latoya] blurts out answers before questions have been asked, does not respond to redirection, and taunts other children whose turn it is to speak, calling them inappropriate names. When you attempt to provide other children with one-on-one attention, [Jake /DeShawn /Emily /Latoya] often disrupts learning by throwing objects and/or bursting into loud laughter. {*On the playground*} [Jake /DeShawn /Emily /Latoya] has been known to act roughly towards other children, sometimes leaving visible scratches {*on their arms, and ignores the rules for safe use of equipment*}. When staff members try to intervene, they scream and run away.

**3. Part B: Parent Vignette**

In Part B, each participant was randomly assigned to receive one of the two parent experimental conditions (either cooperative or uncooperative parents). The child’s name remained identical to the one presented to the teacher in Part A. This was a novel vignette, not used prior to this study.

***3.1. Verbatim Content of the Parent Vignette***

I would like you to imagine that you brought [Jake /DeShawn /Emily /Latoya]’s parents into the classroom so you could speak to them about your concern. You are anxiously waiting to tell [Jake /DeShawn /Emily /Latoya]’s parents. When their parents arrive, they sit down and you begin to tell them about your concerns.

**3.1.1. Cooperative Condition.** [Jake /DeShawn /Emily /Latoya]’s parents immediately acknowledge your concern and ask what steps can be taken so that they can help you work with [Jake /DeShawn /Emily /Latoya].

**3.1.2. Uncooperative Condition.** [Jake /DeShawn /Emily /Latoya]’s parents immediately deny your concern and do not take any recommendations you offer for their child.

**4. Preschool Expulsion Risk Measure (PERM)**

The measures used in the present study are part of the Preschool Expulsion Risk Measure created by Gilliam and Reyes (2018). See “Measures and Analysis Plan” for full details about the measure. Because of the teachers’ busy schedules, we used the two PERM factors (classroom disruption and hopelessness) that were the most relevant to our experimental manipulation and research goals. (For comprehensiveness, we note that the other two factors in the original PERM were: fear of accountability and teacher stress, neither of which were used by Gilliam et al., 2016).

Table S1 shows the patterns for teachers’ ratings on the classroom disruption and feelings of hopelessness after reading about the child (Part A) and after reading about parental cooperativity (Part B), see Figure 1 (main text) for the temporal flow of the study. The correlation matrix in Table S1 shows the associations among the measures, as well as the relevant means, *SD*s, and levels of significance.

**Table S1**

*Preschool Expulsion Risk Measure: Zero-Order Correlations*

| Factors | *M* | *SD* | 1 | 2 | 3 |
| --- | --- | --- | --- | --- | --- |
| Part A |  |  |  |  |  |
| 1. Classroom disruption | 3.71 | 0.90 | – |  |  |
| 2. Hopelessness | 1.68 | 0.85 | .35*** | – |  |
| Part B |  |  |  |  |  |
| 3. Classroom disruption | 3.61 | 0.92 | .84*** | .28*** | – |
| 4. Hopelessness | 1.87 | 0.95 | .19 | .68*** | .18 |

*** *p* < .001

**5. Hopelessness Measure**

As discussed in the main text (“Measures and Analysis Plan”), in our present study, teachers rated three statements to measure their hopelessness about changing the described child’s behavior. These came from the original PERM hopelessness scale (Gilliam & Reyes, 2018) and one of these statements used the word (“help”) that was also used in our experimental manipulation. To be conservative, the main text presented the findings without including teachers’ ratings on the statement that used the word “help.” For completeness, we also re-analyzed the data including the statement with the word “help” thus matching the original full PERM scale. The results found the same significant effects that were reported in the main text: part, *F*(1, 87) = 48.36, *p* < .0001, partial η^2^ = .36; Parental Cooperativity × Part, *F*(1, 87) = 59.55, *p* < .0001, partial η^2^ = .41; and child’s gender, *F*(1, 87) = 4.27, *p* = .042, partial η^2^ = .05. As might be expected, when including the “help” statement, the main effect of parental cooperativity became significant (possibly because the word “help” was repeated), *F*(1, 87) = 7.17, *p* = .009, partial η^2^ = .08, with teachers having higher ratings of hopelessness for the uncooperative (*M* = 2.14, *SD* = 0.65) than the cooperative group (*M* = 1.74, *SD* = 0.78).

**6. Other Teacher-Rated Items**

We preregistered other items for the teachers to rate that we did not analyze in the main paper. For completeness we describe these items here.

In Part A and B, teachers were asked to rate (5-point scale) the degree to which they agreed with the following statement, “this child’s behavior is severe” (from Gilliam et al., 2016). The vast majority (83.16% in Part A; 81.05% in Part B) of teachers agreed that the child’s behavior was “severe” (ratings of 4 or 5). Teachers were also asked if they would recommend disciplinary action for the child. Most teachers (82.11% in Part A; 81.05% in Part B) selected the option that the child’s behavior did *not* merit removal from the classroom. This latter finding is consistent with literature suggesting that removal from the classroom is not the first option for handling child behavior and is often considered only after other attempts to change child behavior were unsuccessful (e.g., DuShane & Yu, 2023; Martin et al., 2018). The severity and disciplinary action variables were not analyzed due to the ceiling effects. We also preregistered three additional items about teachers’ perceptions, but pilot work indicated that these perception items had unacceptable Cronbach’s alpha (< .45), and therefore the items were dropped from the study.

**7. Analyses with Teacher Demographics**

***7.1 Classroom Disruptiveness***

We considered that demographic-related variables may affect teachers’ feelings about children’s classroom disruptiveness, and, thus conducted an analysis of covariance (ANCOVA) controlling for teacher demographics and whether the teacher’s school charged preschool tuition. Specifically, for this analysis, child’s race, child’s gender, and parental cooperativity were the between-subjects factors. Part (A vs. B) was the within-subject factor. The covariates were: (a) teacher race/ethnicity (White vs. non-White), (b) teacher educational level (having a bachelor’s degree vs. not), (c) years of experience in early child education (0, 1, 2, etc.), and (d) preschool tuition (some/all parents paid tuition vs. no parents paid tuition). The ANCOVA (see Table S2) yielded the same pattern of significance as reported in the main text. Two interactions were significant: Parental cooperativity × Part and Child race × Part. No covariates or other variables yielded significant effects, *p*s > .05. In short, the reported effects in the main text remained significant after controlling for teacher demographics and preschool tuition.

**Table S2**

*Repeated-Measures Analysis (ANCOVA) with Outcome of Classroom Disruptiveness*

| Measure | *F*(1, 83) | partial η^2^ |
| --- | --- | --- |
| Within subjects |  |  |
| Part (P) | 0.10 | .00 |
| Teacher race × P | 0.00 | .00 |
| Teacher education × P | 0.02 | .00 |
| Years of experience × P | 1.35 | .02 |
| Tuition × P | 0.51 | .01 |
| Child gender (G) × P | 0.00 | .00 |
| Child race (R) × P | 4.40* | .05 |
| Parental cooperativity (C) × P | 8.51** | .09 |
| G × R × P | 0.31 | .00 |
| G × C × P | 0.93 | .01 |
| R × C × P | 1.30 | .02 |
| G × R × C × P | 1.44 | .02 |
| Between subjects |  |  |
| Covariates |  |  |
| Teacher race | 2.40 | .03 |
| Teacher education | 1.46 | .02 |
| Years of experience | 3.02† | .04 |
| Tuition | 0.04 | .00 |
| Main effects |  |  |
| Child gender | 0.18 | .00 |
| Child race | 1.20 | .01 |
| Parental cooperativity | 0.82 | .01 |
| G × R | 0.82 | .01 |
| G × C | 1.36 | .02 |
| R × C | 0.53 | .01 |
| G × R × C | 0.12 | .00 |

† *p* < .10. **p* < .05. ***p* < .01.

***7.2 Hopelessness***

We also considered that demographic-related variables may affect teacher ratings of hopelessness and conducted an ANCOVA (using the same variables as with classroom disruptiveness in Section 7.1). The ANCOVA yielded significance for two effects: a main effect for child gender and Parental cooperativity × Part (Table S3). No covariates or other variables yielded significant effects, *p*s > .05.

**Table S3**

*Repeated-Measures Analysis (ANCOVA) with Outcome of Hopelessness*

| Measure | *F*(1, 83) | partial η^2^ |
| --- | --- | --- |
| Within subjects |  |  |
| Part (P) | 0.01 | .00 |
| Teacher race × P | 0.17 | .00 |
| Teacher education × P | 0.20 | .00 |
| Years of experience × P | 3.82† | .04 |
| Tuition × P | 1.00 | .01 |
| Child gender (G) × P | 0.09 | .00 |
| Child race (R) × P | 0.99 | .01 |
| Parental cooperativity (C) × P | 15.08*** | .15 |
| G × R × P | 1.57 | .02 |
| G × C × P | 0.03 | .00 |
| R × C × P | 1.52 | .02 |
| G × R × C × P | 0.49 | .01 |
| Between subjects |  |  |
| Covariates |  |  |
| Teacher race | 0.09 | .00 |
| Teacher education | 1.50 | .02 |
| Years of experience | 0.01 | .00 |
| Tuition | 2.46 | .03 |
| Main effects |  |  |
| Child gender | 6.04* | .07 |
| Child race | 0.18 | .00 |
| Parental cooperativity | 0.07 | .00 |
| G × R | 0.32 | .00 |
| G × C | 0.10 | .00 |
| R × C | 0.33 | .00 |
| G × R × C | 0.85 | .01 |

† *p* < .10. **p* < .05. ****p* < .001.

**8. Simplified Factorial Analysis of Variance: Sensitivity Analyses**

Given that the study had a smaller sample size than planned (it was conducted during COVID-19) and was somewhat underpowered for interactions (e.g., four-way interactions), we checked the robustness of the reported findings by conducting additional analyses by collapsing factors while maintaining key manipulation of the study (parental cooperativity vs. non-cooperativity).

More specifically, for the classroom disruption measure, we conducted a 2 (child’s race: Black vs. White) × 2 (parental cooperativity: cooperative vs. uncooperative) × 2 (part: A vs. B) ANOVA (collapsing across child gender, a nonsignificant effect for this measure). The same significant effects reported in the main text remained significant and no additional effects became significant. The results were that the two-way interactions were still significant: Parental cooperativity × Part, *F*(1, 91) = 8.81, *p* = .004, partial η^2^ = .09; Child race × Part, *F*(1, 91) = 5.16, *p* = .025, partial η^2^ = .05.

We followed this same logic for the hopelessness measure. We conducted a 2 (child’s gender: boy vs. girl) × 2 (parental cooperativity: cooperative vs. uncooperative) × 2 (part: A vs. B) ANOVA (collapsing across child race, a nonsignificant effect for this measure). The same significant effects reported in the main text remained significant, and no additional effects became significant. The results were that the main effects were still significant: Part, *F*(1, 91) = 7.73, *p* = .007, partial η^2^ = .08; child gender, *F*(1, 91) = 6.70, *p* = .011, partial η^2^ = .07. Importantly the Parental cooperativity × Part interaction also remained significant, *F*(1, 91) = 17.75, *p* = .000059, partial η^2^ = .16.
